# Optimizing *in vitro* spherulation cues in the fungal pathogen *Coccidioides*

**DOI:** 10.1128/msphere.00679-24

**Published:** 2024-12-17

**Authors:** Christina M. Homer, Elena Ochoa, Mark Voorhies, Anita Sil

**Affiliations:** 1 Division of Infectious Diseases, University of California San Francisco, San Francisco, California, USA; 2 Department of Microbiology and Immunology, University of California San Francisco, San Francisco, California, USA; 3 Chan Zuckerberg Biohub – San Francisco, San Francisco, California, USA; University of Georgia, Athens, Georgia, USA

**Keywords:** *Coccidioides*, fungal pathogenesis, arthroconidia, morphology, development

## Abstract

*Coccidioides* spp. are part of a group of thermally dimorphic fungal pathogens, which grow as filamentous cells (hyphae) in the soil and transform to a different morphology upon inhalation into the host. The *Coccidioides* host form, the spherule, is unique and highly undercharacterized due to both technical and biocontainment challenges. Each spherule arises from an environmental spore (arthroconidium), matures, and develops hundreds of internal endospores, which are released from the spherule upon rupture. Each endospore can then go on to form another spherule in a cycle called spherulation. One of the foremost technical challenges has been reliably growing spherules in culture without the formation of contaminating hyphae and consistently inducing endospore release from spherules. Here, we present optimization of *in vitro* spherule growth and endospore release, by closely controlling starting cell density in the culture, using freshly harvested arthroconidia, and decreasing the concentration of multiple salts in spherulation media. We developed a minimal medium to test spherule growth on various carbon and nitrogen sources. We defined a critical role for the dispersant Tamol in both early spherule formation and prevention of the accumulation of a visible film around spherules. Finally, we examined how the conditions under which arthroconidia are generated influence their transcriptome and subsequent development into spherules, demonstrating that this is an important variable to control when designing spherulation experiments. Together, our data reveal multiple strategies to optimize *in vitro* spherulation growth, enabling characterization of this virulence-relevant morphology.

**IMPORTANCE:**

*Coccidioides* spp. are thermally dimorphic fungal pathogens found in the Southwest United States, Mexico, Central America, and South America. *Coccidioides* can infect both immunocompetent and immunocompromised people and can cause a devastating disseminated infection, including meningitis, with 30% mortality despite all currently available treatments. In this work, we tackle one of the current largest technical barriers to studying the fungus *Coccidioides*: reliably growing its host form *in vitro*. Our work is impactful because we have created a set of foundational tools for the burgeoning field of *Coccidioides* pathogenesis research. We have carefully optimized conditions that allow the development of *Coccidioides in vitro* into its pathogenic form. This work will open up many lines of investigation into the molecules that underlie *Coccidioides* pathogenesis.

## INTRODUCTION

*Coccidioides* spp. are dimorphic fungal pathogens found in the soil in desert regions in North, Central, and South America (1). In the soil, they grow as hyphae and, upon inhalation by a mammalian host, form a unique host-associated morphology known as the spherule (2). Mature spherules are filled with hundreds of endospores and release these internal cells when they rupture. Each endospore can go on to form another spherule in a cycle called spherulation. *Coccidioides* causes infection in immunocompetent and immunocompromised individuals (3), but efforts to develop new treatments and prevention strategies have been hindered by a lack of molecular knowledge of the spherule. This host form of the fungus has been difficult to study due in part to limited *in vitro* culture techniques. Groundbreaking early work by a number of groups established *in vitro* spherulation culture in Converse medium (4–7), Roswell Park Memorial Institute (RPMI) tissue-culture medium (8), and co-culture with HeLa cells (9). Since then, Converse medium has become widely used but the components of this defined medium that are essential for spherulation have not been further identified, and a more minimal medium has not been established. Additionally, since microscopy techniques were limited and quantification was not common, many early reports did not quantify the number of hyphae in spherulation cultures but relied on qualitative metrics. Two reports of continuous pure spherule culture (without hyphae) for 84 days (8) and 4 years (10) were achieved using initially laborious repeated filtration to remove hyphae, which would not be scalable to high-throughput applications. Thus, it remains challenging to grow a pure spherule culture that lacks hyphae, which is necessary to properly model the host phase growth *in vitro*. Finally, it is difficult to consistently induce endospore release without changing media or cell density (11), thereby introducing additional variables into an experiment.

Here, we optimized *in vitro* spherulation, developed a minimal medium for testing which carbon and nitrogen sources can be used for spherule growth, clarified a role for Tamol in dispersing a large visible film surrounding spherules, and developed a modified medium, which consistently induces endospore release from spherules without nutrient supplementation or changing cell density. We additionally found that commonly used storage conditions greatly alter the transcriptome of arthroconidia. Finally, we found that the temperature used to generate arthroconidia affects both their viability and ability to form spherules. Together, this work represents an advance in the ability to consistently produce a pure spherule culture that reliably undergoes endospore release and increases our understanding of the cues required for spherule development.

## MATERIALS AND METHODS

### Strains

The wild-type *Coccidioides posadasii* strain Silveira (NR-48944) was used for all experiments (12).

### Arthroconidia generation

Arthroconidia were inoculated onto 2× glucose yeast extract (GYE) agar [2% dextrose (Fisher), 1% yeast extract (Gibco), and 1.5% bacto-agar (BD)] + 1× penicillin/streptomycin (100 U/mL penicillin and 100 g/mL streptomycin, UCSF Media Core) in T225 tissue culture flasks and grown for 4–6 weeks at 30°C, until the hyphal mat appeared dry and flattened as previously described (11). Arthroconidia were harvested 0–2 days prior to initiating spherulation and stored at 4°C until use unless noted in the text. The arthroconidia harvest was done as previously described (11), by adding phosphate buffered saline (PBS, UCSF Media Core) to tissue culture flasks with the hyphal mat, scraping to resuspend, and filtering through a 70-micron mesh filter. Arthroconidia were then washed twice with PBS and resuspended in PBS at appropriate concentrations for downstream assays. Arthroconidia were quantified using a plastic hemacytometer (Neubauer, C-Chip) sealed with nail polish.

### Microscopy of spherulation and hyphal growth

Standard spherulation conditions: 125 mL polypropylene flasks containing 50 mL of Converse medium inoculated with 10^6^arthroconidia/mL arthroconidia (unless otherwise stated) and placed at 39°C, 10% CO_2_, shaking at 120 rpm. Converse medium was made as previously published (11) except for alteration in Tamol concentration [0.016 M NH_4_CH_3_CO_2_ (Sigma Aldrich), 0.0037 M KH_2_PO_4_ anhydrous (Fisher), 0.003 M K_2_HPO_4_ anhydrous (Fisher), 0.0016 M MgSO_4_*7H_2_O (Fisher), 1.25 × 10^−5^ M ZnSO_4_*7H_2_O (Fisher), 2.4 × 10^−4^ M NaCl (Fisher), 2.04 × 10^−5^ M CaCl_2_*2H_2_O (Fisher), 1.43 × 10^−4^ M NaHCO_3_ (Fisher), 0.5% Tamol (Northeast Laboratory), 0.4% glucose (Fisher), and 0.5% N-Z amine (Fisher)]. All stocks/media were sterile filtered using a 0.45-micron vacuum filter instead of being autoclaved. Where noted, alterations were made to the Converse medium including removing/reducing concentrations of individual components. Additional ingredients used in Converse alterations are as follows: 0.016 M KCH_3_CO_2_ (Sigma Aldrich), 0.016 M NaCH_3_CO_2_ (Fisher), 0.016 M NH_4_Cl (Fisher), 0.016 M (NH_4_)_2_SO_4_ (Molecular Sigma), 0.016 M NH_4_HCO_3_ (Sigma Aldrich), and supplemental 0.016 M NaHCO_3_ (Fisher). For hyphal growth, 125-mL polypropylene flasks containing 50 mL of Converse medium were inoculated with 10^6^ arthroconidia/mL and grown at 25°C shaking at 120 rpm. At the stated timepoints for light microscopy (day 2–3, day 4–5, and day 7 post-inoculation), cells were fixed with 4% paraformaldehyde (PFA) (Electron Microscopy Sciences) at room temperature for 30 minutes and washed twice in PBS, pelleting cells by centrifugation for 2 minutes at maximum speed between washes. Cells were visualized using a 40× DICII objective on a Zeiss Axiovert 200 microscope, with additional 1.6× Optovar magnification. All images presented are representative of at least two independent biological replicates.

### Making endoConverse medium

Using Converse medium as described above, the pH was first raised to 11 with 4 N NaOH and then lowered to 8.5 with 6 N HCl. Visible precipitate was observed and then removed by sterile filtration with a 0.45-micron filter.

### Identifying precipitate produced during generation of endoConverse medium

Using 100 mL of normal Converse, the pH was increased to 11 with 4 N NaOH and then lowered to 8.5 with 6 N HCl. The precipitate was pelleted by centrifugation (maximum speed, 5 minutes) and the supernatant was removed. The precipitate was then sent to EAG Laboratories and analyzed by Scanning Electron Microscope/Energy Dispersive X-Ray Spectroscopy. Results presented in Fig. 3C are from EAG Inc. (www.eag.com).

### Arthroconidia aging

For the experiment described in Fig. 5, arthroconidia were harvested as described above and placed at 4°C in PBS (UCSF Media Core) in the dark. At specified timepoints, 3 aliquots were removed from the stock and RNA was immediately harvested as described below.

### Generating arthroconidia at different temperatures

For the experiment described in Fig. 6, arthroconidia were generated at either 25°C, 30°C, or 37°C for 4 weeks and then harvested as described above. Three aliquots were removed from the arthroconidia stock, and RNA was immediately harvested as described below. This experiment was repeated for a total of two replicates per stock generated at each temperature. One of the biological replicates harvested at 30°C is the same sample that was used for week 0 in the arthroconidia aging experiment in Fig. 5.

### RNA extraction and RNA-seq library preparation

RNA from arthroconidia were collected from the same arthroconidia stock in triplicate by placing 5 × 10^7^ arthroconidia into TRIzol LS (Ambion) and bead beating for 2 minutes. Samples were stored at −80°C until samples from all timepoints in an individual experiment had been collected. RNA was extracted using the Direct-zol RNA Miniprep Plus Isolation Kit (Zymo) with the on-column DNAse digestion step extended for 15 minutes. Sequencing libraries were prepared using the Ultra II Directional RNA Library Prep Kit with dual-indexed multiplexing barcodes, with total RNA as the input, omitting the polyA selection step. Library quality and adaptor dimer contamination were analyzed using Agilent High Sensitivity DNA Chip. An additional round of library size selection was performed using homemade Serapure size selection beads (13) for libraries containing significant adaptor dimers. Final library concentrations were measured using the Qubit High Sensitivity or Broad Range reagents. Libraries were pooled and sequencing was then performed on three and two lanes of the Novaseq 6000 S4 at the Chan Zuckerberg Biohub, San Francisco, for Fig. 5 and 6, respectively.

### RNA-seq data analysis

Analysis was conducted as previously described (14) with alterations below. Briefly, estimated counts of each transcript were calculated for each sample by alignment-free comparison against the predicted mRNA for the published Silveira genome (12) using KALLISTO version 0.46.2 (15). Further analysis was restricted to transcripts with raw counts ≥ 10 in at least one sample across an individual experiment. Differentially expressed genes were identified by comparing replicate means for contrasts of interest using LIMMA version 3.46.0 (16). Genes were considered significantly differentially expressed if they were statistically significant (at 5% false discovery rate) with an absolute log_2_ fold change ≥ 1 for a given contrast unless otherwise noted in the text. The Euler diagram in Fig. 6 was generated using the eulerAPE package version 3.0.0 (17).

### Measurement of arthroconidia viability

Arthroconidia viability was measured using two different methods: (i) arthroconidia stocks were diluted 1:100 in 0.4% trypan blue (Sigma) and counted on a hemacytometer. Arthroconidia were classified as viable if they excluded the blue dye and were classified as non-viable if they were blue. (ii) Dilutions of arthroconidia stocks were plated on 2× GYE agar + penicillin/streptomycin, incubated at 30°C for 72 h, and colonies were counted. Percent viability was defined as the number of colonies that had grown out of the total number of arthroconidia plated (by hemacytometer counts).

## RESULTS

### *In vitro* spherule formation depends on arthroconidia concentration

**Fig 1:**
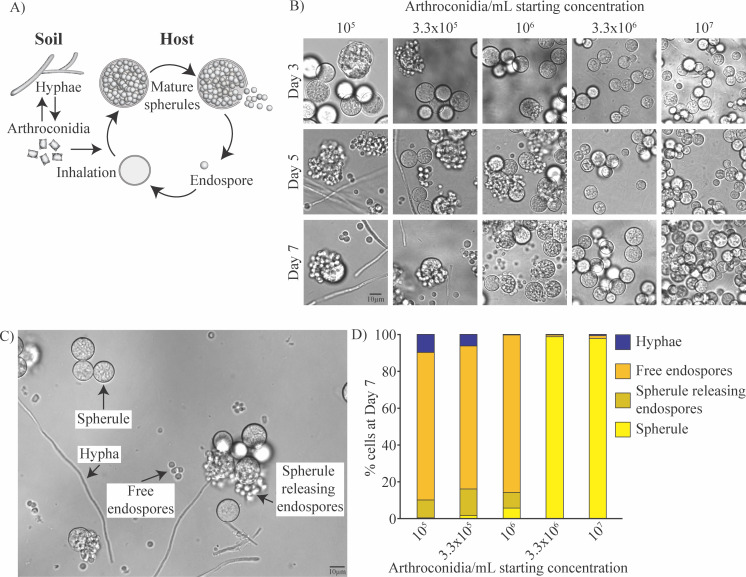
Optimizing starting concentration of arthroconidia for *in vitro* spherule formation. (A) The schematic depicts the *Coccidioides* lifecycle. Hyphae grow in the soil and produce infectious spores named arthroconidia. If inhaled, arthroconidia enlarge into spherules that partition internally, generating endospores that are released when the spherule ruptures. Released endospores develop into new spherules and continue the cycle of spherulation. (B) Different concentrations of arthroconidia ranging from 10^5^ to 10^7^ arthroconidia/mL were placed in Converse medium + 0.5% Tamol. Cells were fixed with 4% PFA and monitored by light microscopy at days 3, 5, and 7 post-inoculation and monitored for spherule formation, hyphae formation, and endospore release. At higher densities, hyphae formation was suppressed but spherules did not release endospores during the experiment. At lower concentrations, there was more hyphal contamination of cultures but spherules did release endospores. (C) Differential interference contrast (DIC) microscopy image of a spherulation culture fixed in 4% PFA demonstrating all four possible morphologies for quantification schema: spherule prior to endospore release, spherule in the process of releasing endospores, which are still associated with the spherule form, free endospores that have disassociated from the spherule that released them, and hyphae. (D) Quantification of the proportion of each morphologic form in cultures on day 7 from B, using the schema demonstrated in C. Each morphology was quantified by hand for at least 10 fields of view for each sample.

To study spherule morphology in detail, our first goal was to reproducibly generate spherules without hyphae also developing in the same culture. Arthroconidia germinate and develop into spherules, which go on to release endospores (Fig. 1A). We tested spherule formation by placing arthroconidia in media known to induce spherules [RPMI + 10% fetal bovine serum (FBS) (8, 11) or Converse medium (4–7)] and germinated them in standard spherule-inducing conditions (39°C, 10% CO_2_). We added 0.5% Tamol, a chemical dispersant that was shown to promote spherule growth and aid uniform diffusion of clumped endospores after release from spherules (5) to Converse medium to encourage optimal growth(4) (referred to as Converse medium hereafter). Additionally, we placed arthroconidia in medium similar to that used for macrophage infections with bone marrow-derived macrophages [Dulbecco’s modified Eagle medium (DMEM) + 20% FBS (18–20)] and germinated them in spherule-inducing conditions. While spherules formed and released endospores in all three conditions (Fig. S1), arthroconidia placed in Converse medium formed consistent spherules with very few hyphae so we chose this as our base medium for further study. Next, given precedent that cell density can influence spherule formation and purity of spherule cultures (8), we assayed whether cell density impacted the rate of spherule formation, the amount of hyphae in spherule cultures, and endospore release. We placed freshly harvested arthroconidia of varying densities in Converse media, germinated them in standard spherule-inducing conditions, and monitored spherule formation by light microscopy (Fig. 1B). Lower density spherulation cultures were comprised of spherules, the vast majority of which went on to release endospores (quantification schema in Fig. 1C, quantification data Fig. 1D). Higher density cultures formed spherules, but those spherules did not go on to release endospores when the 7-days culture were observed, perhaps due to nutrient depletion, accumulation of a metabolite that inhibits endospore release, or cell density-dependent signaling. We observed more hyphae in lower density cultures than higher density cultures. It is not clear whether those hyphae originated from newly released endospores or from the original arthroconidia, although hyphae generally appeared after endospore release. Cultures started at a concentration of 10^6^ arthroconidia/mL in Converse medium and germinated under standard spherulation conditions released endospores starting on day 3 and produced minimal numbers of hyphae. Therefore, we chose this as our standard arthroconidia concentration for starting spherule cultures.

### Developing a minimal medium for spherulation

**Fig 2:**
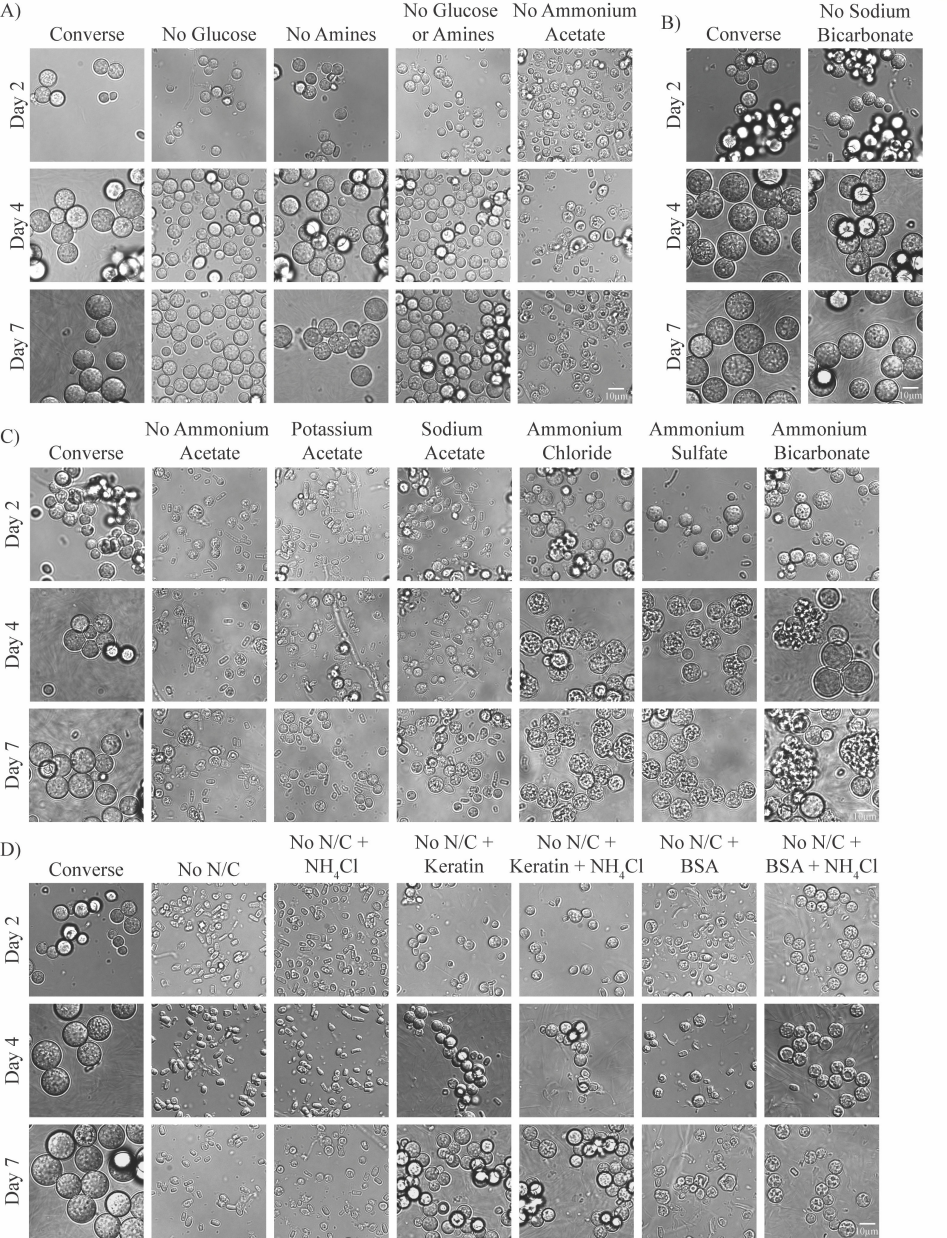
Defining carbon and nitrogen sources during spherulation. 10^6^ arthroconidia/mL were grown in standard spherulation conditions (39°C, 10% CO_2_) in media variations described below, and the resultant cells were fixed with 4% PFA and monitored for spherule formation by light microscopy on days 2, 4, and 7 post-inoculation. (A) Media were either standard Converse or Converse lacking glucose (No Glucose), lacking N-Z amines (No Amines), lacking both glucose and N-Z Amines (No Glucose or Amines), or lacking ammonium acetate (No Ammonium Acetate). (B) Media were either standard Converse or Converse lacking sodium bicarbonate. (C) Media were either standard Converse, Converse lacking ammonium acetate (No Ammonium Acetate), or Converse in which the ammonium acetate is replaced by the same concentration of potassium acetate, sodium acetate, ammonium chloride, ammonium sulfate, or ammonium bicarbonate. (D) Media were either Converse or No N/C (Converse medium lacking carbon and nitrogen sources: glucose, N-Z amines, ammonium acetate, and sodium bicarbonate), supplemented with 3% keratin, 3% BSA, and/or 0.016 M NH_4_Cl. The biological replicate labeled as “Converse” is the same biological replicate as presented in B.

We wanted to create a minimal medium for spherulation that could be used to test the effect of various carbon sources. Converse has four ingredients that are standard carbon sources: glucose, amines, ammonium acetate, and sodium bicarbonate. The chemical Tamol (5) also contains carbon although, to our knowledge, it is not known whether organisms are able to use it as a carbon source. We placed arthroconidia in one of the following (Fig. 2A and B): (i) Converse; (ii) Converse lacking glucose, amines, or ammonium acetate; (iii) Converse lacking glucose and amines; or (iv) Converse lacking sodium bicarbonate. Spherules developed in all these media variations except for those lacking ammonium acetate, in which some cells appeared to be arrested at the stage of isotropic swelling or early spherule initials, in combination with ungerminated arthroconidia. Despite using optimized spherule conditions determined above, endospore release was not consistent (Converse in Fig. 2A through D compared with Fig. 1B), likely due to variation in starting arthroconidia stock viability, which changes the effective starting arthroconidia concentration. Of note, the spherule diameter in media lacking glucose was smaller than those of spherules in normal Converse or Converse lacking amines (Fig. S2A). The spherule diameter in media lacking sodium bicarbonate varied significantly between replicates (not shown), so we cannot conclude that it changes the spherule diameter consistently compared with normal Converse. Since ammonium acetate could be promoting growth by serving as either a carbon source (acetate) or a nitrogen source (ammonium), we substituted other sources of acetate or ammonia for ammonium acetate and found that the growth defect in Converse lacking ammonium acetate could be partially or fully rescued by other chemicals containing ammonia (Fig. 2C), indicating that ammonia appears to be the critical nitrogen source during spherulation despite the presence of amines in the media. As spherules grown in media containing ammonium chloride and ammonium sulfate instead of ammonium acetate are smaller than spherules in normal Converse, there is likely some role for acetate as a carbon source. Consistent with that, spherules grown in Converse containing ammonium bicarbonate instead of ammonium acetate grew to a similar size as spherules grown in Converse with ammonium acetate (Fig. 2C) and Converse containing ammonium chloride or ammonium sulfate instead of ammonium acetate and then supplemented with additional sodium bicarbonate also grew to a similar size as spherules grown in normal Converse (Fig. S2B). This is likely because bicarbonate can also serve as a carbon source, like acetate.

Since our data indicate that spherules can use multiple carbon sources, we created a Converse medium lacking glucose, amines, ammonium acetate, and sodium bicarbonate. As expected, arthroconidia placed into this medium in spherulation conditions were not able to form spherules (and also do not form hyphae), indicating they were not able to germinate and grow (Fig. 2D). Since we know ammonium acetate serves as an important nitrogen source as well, we added ammonium chloride to this medium and found that it did not restore growth, suggesting the lack of growth is due to the lack of carbon availability, despite Tamol being present in the media. Therefore, we conclude Tamol does not serve as a sufficient carbon source during spherulation. Finally, to demonstrate that this “no-carbon” Converse can be used to test the efficacy of various carbon sources for spherulation, we added keratin or BSA to “no-carbon” Converse with or without ammonium chloride to see if *Coccidioides* spherules are able to use these proteins as carbon sources. We observed small spherules forming in the media containing keratin with or without ammonium chloride supplementation, indicating that keratin can serve as both a carbon and a nitrogen source during spherulation (Fig. 2D; Fig. S2C). However, in “no carbon” Converse supplemented with BSA, we only observed small misshapen forms, potentially spherule initials, that were not able to progress through development. When “no-carbon” Converse with BSA was supplemented with additional nitrogen (ammonium chloride), small spherule initials were able to form although they had a different appearance with more accentuated septations than conditions containing keratin or normal Converse. The significance of this difference in appearance is not clear. Together, these data suggest *Coccidioides* spherules can use keratin as both a carbon and nitrogen source and potentially BSA as a suboptimal carbon source. The keratin findings are particularly intriguing given that (i) *Coccidioides* is enriched in the environment in rodent burrows (21, 22), which are abundant in keratin, and (ii) clinically, disseminated *Coccidioides* infections often involve the skin, which is a keratinous structure (23).

### Optimizing *in vitro* endospore release

**Fig 3:**
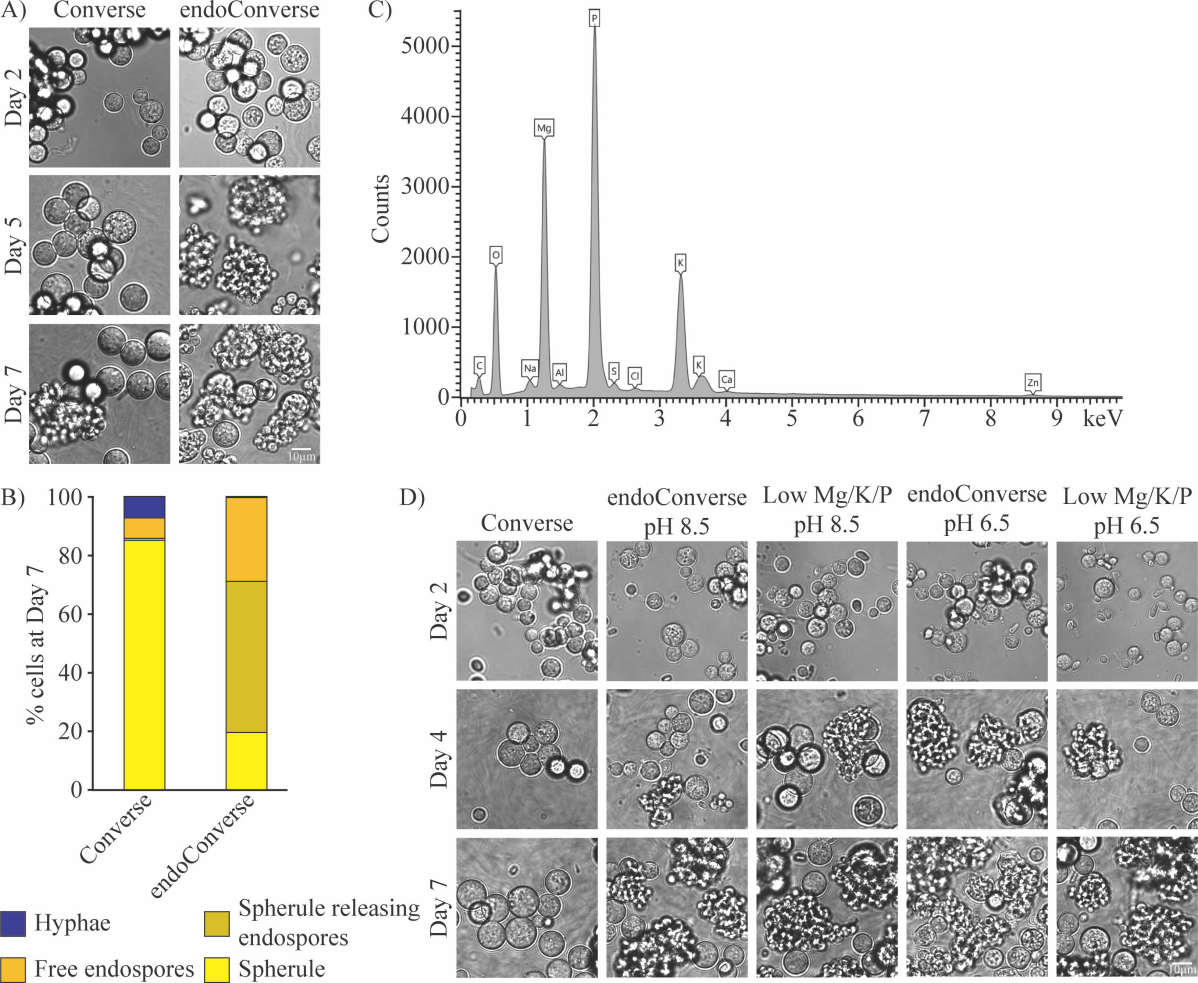
Optimizing endospore release. (A) 10^6^ arthroconidia/mL were grown in standard spherulation conditions (39°C, 10% CO_2_) in either Converse or endoConverse. Cells were fixed with 4% PFA and monitored by light microscopy at days 2, 5, and 7 post-inoculation for spherule formation. (B) Quantification of the proportion of each morphology in cultures on day 7 from 3A (schema in 1C). Each morphology was quantified by hand for at least five fields of view for each sample. (C) Identification of precipitate removed in the process of making endoConverse by energy-dispersive X-ray spectroscopy. (D) 10^6^ arthroconidia/mL were grown in standard spherulation conditions (39°C, 10% CO_2_) in either Converse or endoConverse with starting pH 6.5 or 8.5 and Converse medium with only 10% the normal starting amount of MgSO_4_, KH_2_PO_4_, and K_2_HPO_4_. Cells were fixed by 4% PFA and monitored by light microscopy at days 2, 4, and 7 post-inoculation for spherule formation. The images labeled as “Converse” are the same biological replicate and images as presented in 2C.

Endospore biology, including endospore release, has not been studied systematically, and the molecular determinants of endospore release are unknown. To robustly study this part of the spherulation cycle, we wanted to develop *in vitro* conditions where endospore release occurs reliably since we had observed differences in the quantity of endospore release even in our optimized Converse conditions. While many of our samples in Converse medium in spherulation conditions release endospores between days 3 and 7, the degree of endospore release is variable and spherules releasing endospores can be a small percentage of the culture in some instances. We found that raising the pH of Converse to 11 causes a precipitate to form, and when that precipitate is removed through filtration and the pH adjusted to 8.5, the resulting medium (which we call endoConverse) induces a dramatically increased amount of endospore release by day 5 (Fig. 3A), with the vast majority of spherules releasing endospores on day 7 (Fig. 3B). We identified the elemental components of this precipitate using energy-dispersive X-ray spectroscopy. While this assay is not quantitative, it can distinguish major and minor components. The assay identified that the major components of the precipitate were phosphorus, magnesium, potassium, and oxygen (Fig. 3C). Based on the ingredients present in Converse media, we hypothesize that the magnesium sulfate and potassium phosphate are combining to form magnesium potassium phosphate salt, which has been shown to precipitate at high pH (24). We attempted to replicate this effective decrease in salts via precipitation by reducing the MgSO_4_, KH_2_PO_4_, and K_2_HPO_4_ to 10% of their amount in normal Converse and adjusted the pH of that medium to 8.5 to match endoConverse. When arthroconidia were placed into this altered medium and grown under standard spherulation conditions, they did have significantly increased endospore release compared with normal Converse (Fig. 3D). In fact, this effect held when the starting pH was 6.5 as well, as in normal Converse. Further investigation is warranted into the endospore release signaling pathways that are inhibited by the Mg, K, P, O-containing salt, which may give insight into when and how endospore release is triggered during infection.

### Tamol dislodges a visible outer film from spherules

**Fig 4:**
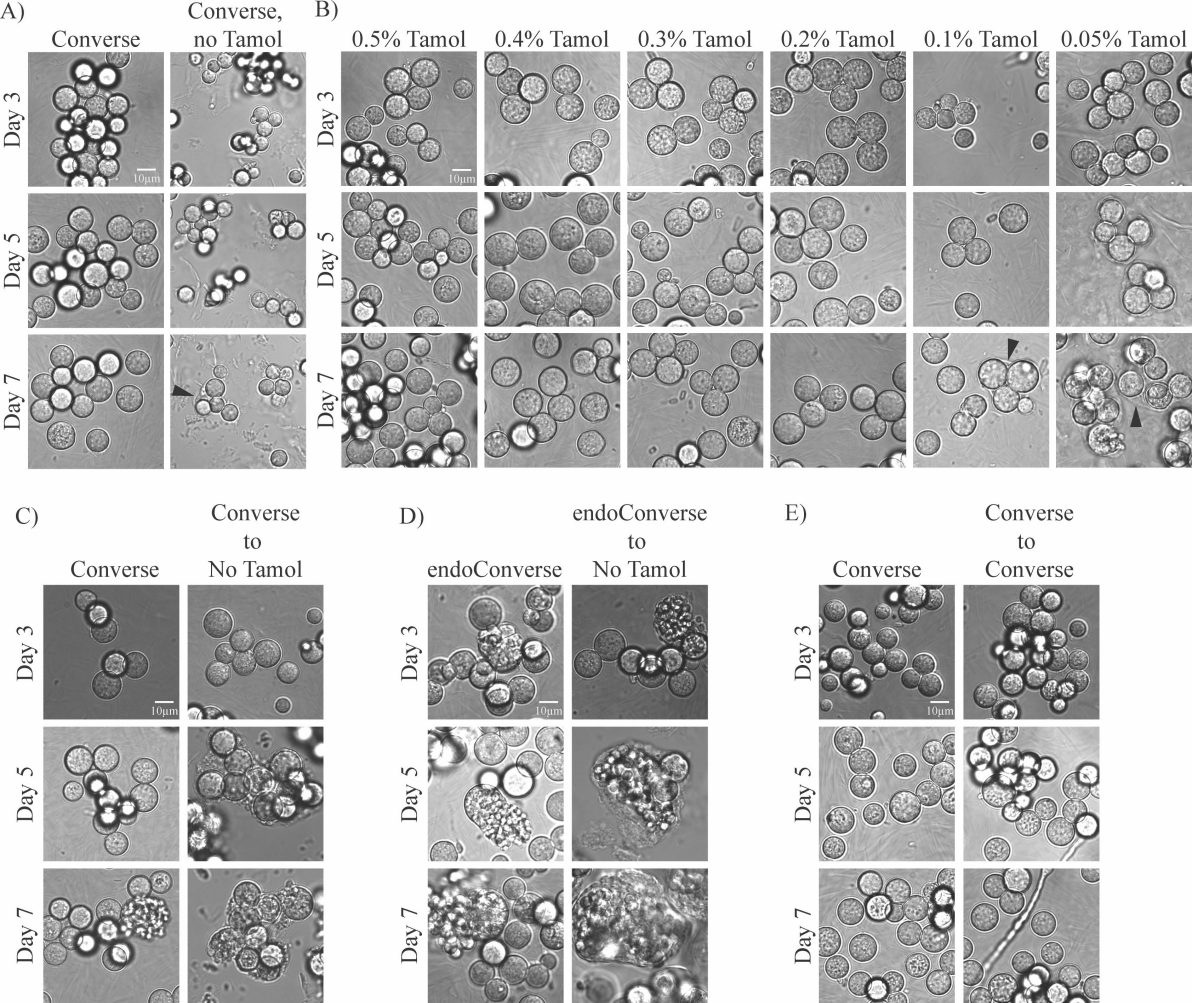
Dissecting the role of Tamol in spherulation. 10^6^ arthroconidia/mL were grown in standard spherulation conditions (39°C, 10% CO_2_) in the media variations below. Cells were fixed with 4% PFA and monitored by microscopy at days 3, 5, and 7 post-inoculation for spherule formation. (A) Media were either Converse or Converse without Tamol. (B) Media were either Converse or Converse with decreasing amounts of Tamol. The biological replicate labeled as “0.5% Tamol” is the same biological replicate as that labeled as “Converse” in A. Black arrowheads point to the layer observed to accumulate around groups of spherules in the lowest concentrations of Tamol tested. (C) The medium was Converse at initiation of spherulation. On day 3, after cells were visualized by microscopy, samples labeled “Converse to No Tamol” were spun down at 2,500 rpm for 5 minutes, supernatant was discarded, and cells were resuspended in 50 mL of Converse lacking Tamol. They were then replaced at incubation conditions. (D) Same as in C but spherules initiated with endoConverse instead of standard Converse medium and exchanged to Converse lacking Tamol on day 3. (E) Same as in C but exchanged to standard Converse on day 3.

As Tamol is an unusual ingredient in growth media, we interrogated the role it plays in Converse media. First, we eliminated Tamol entirely from Converse and found that, while arthroconidia placed in spherulation conditions still formed small spherules, their growth was arrested compared with the same medium with Tamol, and these small spherules did not go on to release endospores (Fig. 4A). As Tamol was not found to be a sufficient carbon or nitrogen source (Fig. 2), we do not think Tamol is altering the size of spherules due to nutritional factors. Instead, we hypothesize that its characteristics as a dispersant are altering the spherule surface in some way that facilitates expansion of the spherule cell wall. Next, we queried a range of Tamol concentrations in Converse (from 0.05% to 0.5%) and found that even the lowest concentrations of Tamol were sufficient to form spherules that are a similar size to those grown in Converse with 0.5% Tamol (Fig. 4B), although only a very small amount of endospore release was observed in these flasks. However, at the two lowest concentrations of Tamol tested (0.05% and 0.1%) and in Converse lacking Tamol, we observed accumulation of a substance around multiple spherules (arrowheads). This film is larger and more pronounced in the lowest concentration of Tamol tested. To further evaluate this film, we induced spherulation in normal Converse or endoConverse and then shifted already-formed spherules on day 3 into Converse without Tamol. In these cultures, clumps of spherules or clumps of spherules releasing endospores accumulated a large, highly visible layer of this substance around themselves (Fig. 4C and D). This is not an effect of the media exchange, as transferring spherules into Converse with 0.5% Tamol on day 3 does not cause a similar effect (Fig. 4E). While we have not characterized this film definitively, we hypothesize that it is the spherule outer wall (SOW), which has been previously identified as a structure that is shed into the media of spherulating cultures (25), and that Tamol is increasing the rate of that shedding. Therefore, in cultures with low or no Tamol, the rate of SOW shedding is decreased to the point that it accumulates around spherules into a thick-enough film to be visible by microscopy. Further work is necessary to confirm whether this accumulating layer is the SOW and whether such a layer accumulates during infection in a mammalian host.

### Time in storage conditions greatly alters the transcriptome of arthroconidia and affects *in vitro* spherule formation

**Fig 5:**
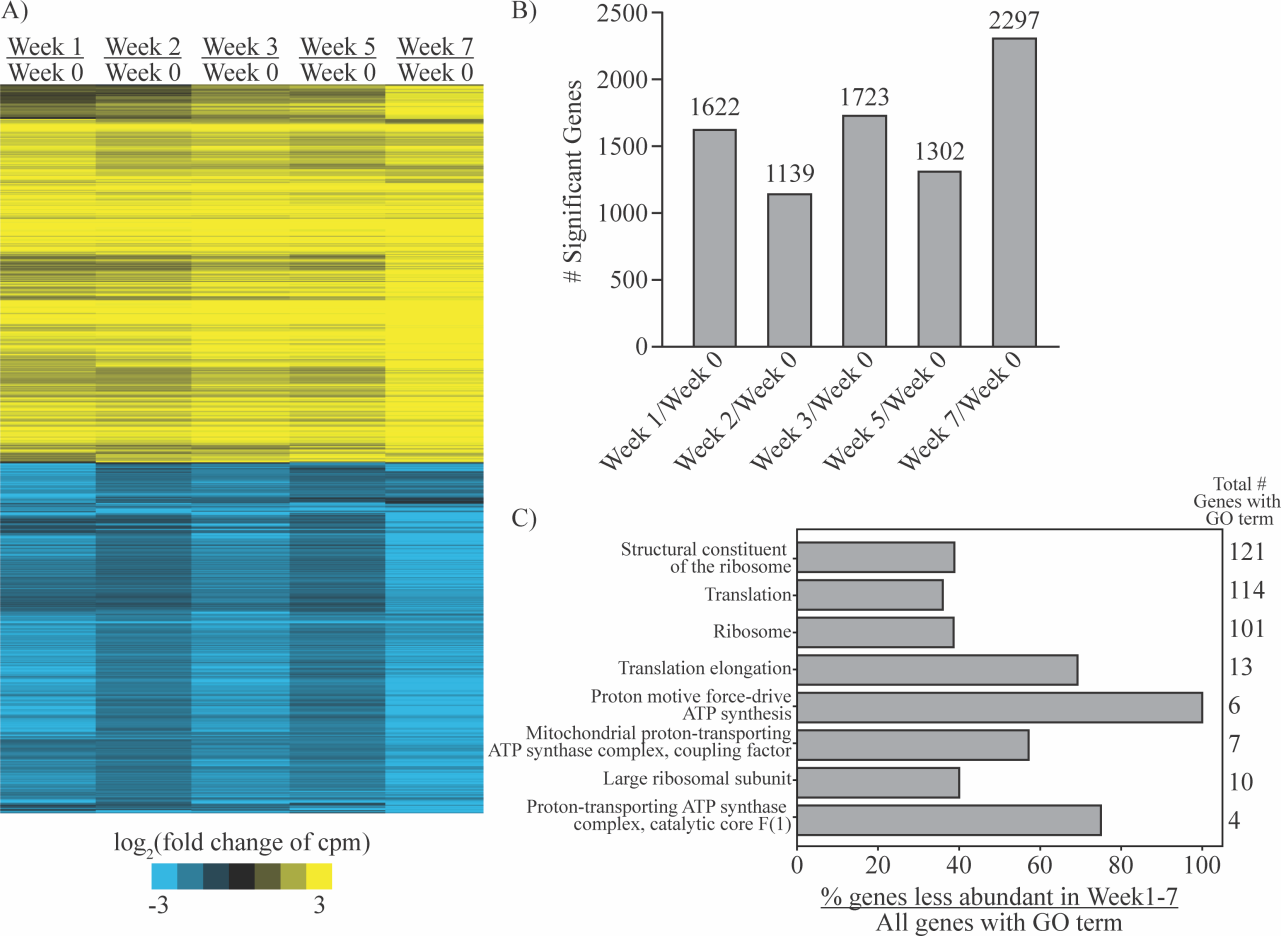
Storing arthroconidia in PBS at 4°C induces large-scale transcriptome changes. (A) Heatmap demonstrating comparisons of the transcriptome between a stock of freshly harvested arthroconidia and arthroconidia stored at 4°C in PBS for 1, 2, 3, 5, and 7 weeks after harvest. Yellow (positive) and blue (negative) shades represent log_2_ fold change values of counts per million (cpm) for each stated comparison. Each row represents one transcript, and rows were clustered based on behavior across all comparisons. (B) Bar graph showing the number of significantly differentially regulated transcripts (fourfold change, FDR 5% in limma) for each comparison of triplicate samples at each stated timepoint. (C) Percentage of genes with lower abundance in storage conditions that were associated with a particular Gene Ontology (GO) term, with total genes in the genome associated with that same GO term labeled on right of graph.

The current standard method of storing *Coccidioides* arthroconidia stocks is in PBS at 4°C. We interrogated whether storage in these conditions alters the propensity of the arthroconidia stocks to form spherules when placed in our optimized spherulation conditions. Indeed, we found that the longer arthroconidia stocks were stored at 4°C in PBS, the more hyphae were present in spherule cultures generated from those stocks (Fig. S3A). Given the precedent that the transcriptome of a fungal spore influences cellular phenotypes after spore germination (26, 27), we assayed the transcriptome of an arthroconidia stock after it was freshly harvested or over time in storage. For stored arthroconidia stocks, we placed arthroconidia directly from PBS at 4°C into TRIzol so that they did not have an opportunity to germinate. An extremely high number of transcripts changed in abundance during storage compared with freshly harvested arthroconidia; therefore, we chose a fourfold cutoff instead of the conventional twofold cutoff to emphasize transcripts that were most differential. Indeed, we found that 2,559 transcripts demonstrated a significant change in abundance between at least one timepoint in storage compared with freshly harvested arthroconidia (fourfold cutoff, false discovery rate [FDR] 5%) (Fig. 5A; Fig. S3B; Table S1). There were similar numbers of transcripts whose abundance increases versus decreases during storage. Storage for 1–5 weeks at 4°C in PBS induced significant differences in transcript abundance for 1,139 to 1,723 transcripts (Fig. 5B), and storage for 7 weeks induced the most change in transcript abundance (2,297 transcripts). Overall, there was a similar trend in magnitude and direction of change for all transcripts responsive to placement in storage conditions.

While no clear Gene Ontology (GO) term enrichment was found in transcripts whose abundance was increased upon storage, some transcripts related to sexual development and meiosis are, surprisingly, more abundant in 4°C storage conditions, including D8B26_004117 [ortholog of *Saccharomyces cerevisiae*’s *DMC1* meiotic recombinase (28)], D8B26_000913 [ortholog of *VELC*, a regulator of sexual development in *Aspergillus nidulans* (29) and *Aspergillus fumigatus* (30)], D8B26_006129 [ortholog of *SPO11* in *S. cerevisiae* (31), a meiosis-specific topoisomerase], D8B26_004631 [ortholog of *CSM3* in *S. cerevisiae* (32, 33), which plays a role in chromosome segregation during meiosis], D8B26_001535 [ortholog of *HOP1* in *S. cerevisiae* (34), where it plays a role in homolog pairing during meiosis], and D8B26_04250 [ortholog of *MND1* in *S. cerevisiae* (32, 35), where it plays a role in recombination and meiotic nuclear division]. Examining the 264 transcripts that have significantly decreased abundance at all subsequent timepoints compared with week 0, transcripts with GO terms related to the ribosome or translation are significantly enriched (Fig. 5C). Given many of these less abundant transcripts are structural constituents of the ribosome, this likely implies a decrease in steady-state levels of ribosomes, but it remains to be explored whether this is due to a decrease in production of ribosomes or an increased rate of ribosomal transcript degradation. Almost all the genes containing GO terms related to ATP synthase are also less abundant upon storage. This may reflect a shift in energy sources or possibly a shift into dormancy, as is expected in a number of spore types (26, 36, 37).

### Generating arthroconidia at different temperatures affects their transcriptome and behavior upon germination

**Fig 6:**
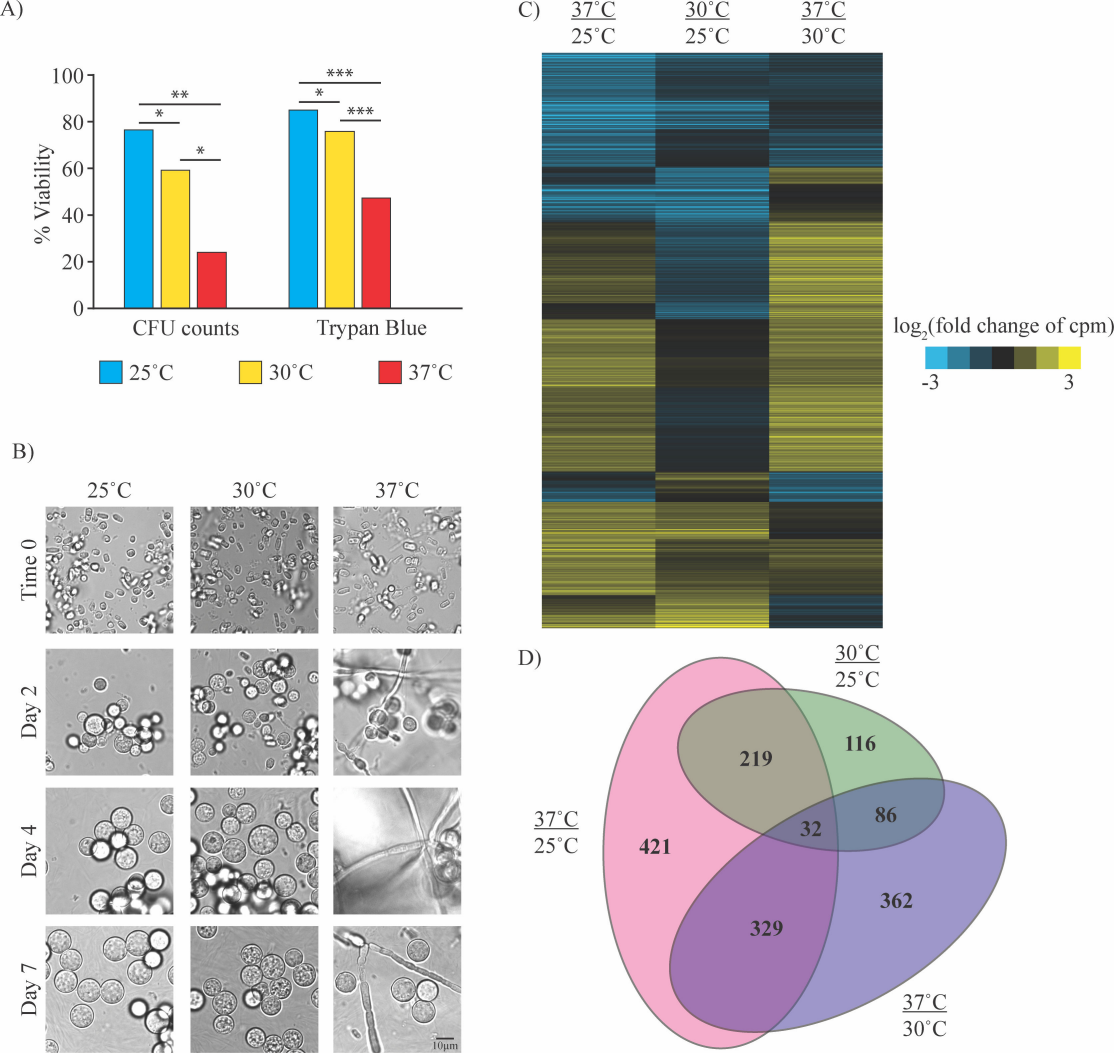
Generating arthroconidia at different temperatures affects their transcriptome and behavior upon germination. (A) Percentage of viable arthroconidia assessed by trypan blue exclusion (on day of arthroconidia harvest) or by CFU counts (after growth for 72 h on 2× GYE at 30°C). *p < 0.05, **p < 0.005, and ***p < 0.0005 by paired t-test. (B) 10^6^ viable arthroconidia/mL (as assessed by trypan blue), generated at either 25°C, 30°C, or 37°C, were grown in standard spherulation conditions (39°C, 10% CO_2_) in Converse media. Cells were fixed with 4% PFA and monitored by light microscopy on days 2, 4, and 7 post-inoculation for spherule and hyphae formation. (C) Heatmap demonstrating comparisons of the transcriptome between arthroconidia stocks generated at 25°C, 30°C, and 37°C. Yellow (positive) and blue (negative) shades represent log_2_ fold change values of counts per million for each stated comparison. Each row represents one transcript, rows were clustered based on behavior across all comparisons. (D) Euler diagram demonstrating overlap between differential transcripts for comparisons between arthroconidia stocks generated at different temperatures.

Given that storage conditions affected the transcriptome of arthroconidia, we also sought to determine whether the conditions used to generate arthroconidia affected their transcriptome and behavior after germination. We generated arthroconidia by growing hyphal mats for 4 weeks at either 25°C, 30°C, or 37°C. After harvesting, we tested the viability of these arthroconidia stocks by trypan blue exclusion or quantifying the number of CFUs generated by each stock (Fig. 6A; Fig. S4A demonstrating additional replicate given variability in this assay). While viability measured by CFUs is consistently lower than by trypan blue, both methods indicate decreased viability of the arthroconidia stocks generated at 37°C compared with stocks generated at other temperatures. We then went on to characterize the ability of these arthroconidia stocks to generate spherules. Given our previous observations that lower concentrations of arthroconidia generate spherulation cultures containing more hyphae, we attempted to compensate for the decreased viability of arthroconidia generated at 37°C by inoculating with 10^6^ viable arthroconidia as assessed by trypan blue exclusion (since that measure of viability is available at the time of spherule culture initiation). Despite this compensation, spherulation cultures initiated with arthroconidia generated at 37°C had significantly more hyphae than arthroconidia stocks generated at 25°C and 30°C (Fig. 6B). This may be because trypan blue staining overestimates arthroconidia viability (e.g., as compared with viability measurements by CFUs). However, the more intriguing possibility is that the conditions under which arthroconidia are generated are affecting spherulation behavior. Further study will be needed to verify this effect.

To delve deeper into the differences caused by generating arthroconidia at various temperatures, we assayed the transcriptome of these arthroconidia stocks, placing arthroconidia directly from the PBS in which they were harvested into TRIzol so that they did not have the opportunity to germinate. We found that 1,001 transcripts were significantly changed when comparing arthroconidia generated at 37°C versus 25°C. As expected, slightly fewer transcripts were significantly different when comparing the intermediate 30°C temperature: 809 transcripts were significantly differential between arthroconidia generated at 37°C versus 30°C, and 453 transcripts were significantly differential between arthroconidia generated at 30°C versus 25°C (twofold cutoff, FDR 5%, Fig. 6C and D; Table S2). Indeed, the larger number of significantly differential transcripts in comparisons involving the 37°C-generated stock correlates with the more significant phenotypes of that arthroconidia stock (viability defect and hyphal contamination in spherule cultures). When focusing on the most drastic comparison of 37°C-generated arthroconidia compared with 25°C-generated arthroconidia, >50% of transcripts with the GO term 6270 (DNA replication initiation) are increased in abundance in the 37°C-generated arthroconidia, including D8B26_002886 [the ortholog of *CDC45* in *S. cerevisiae*, a DNA replication initiation factor (38)], D8B26_000131 [the ortholog of *SLD2*, which is required for chromosomal replication in *S. cerevisiae* (39)], D8B26_008289 [the ortholog of *CDC6*, a protein required for DNA replication initiation (40, 41) and that has been shown to interact with the eukaryotic replicative helicase in *S. cerevisiae* (42)], and multiple components of the eukaryotic replicative helicase (D8B26_000480, D8B26_006296, and D8B26_004943). The significance of increased abundance of transcripts encoding replication factors in these presumably dormant arthroconidia is unknown and may suggest dysfunctional regulation of DNA synthesis in the 37°C-generated arthroconidia stocks that impacts their viability and potential to germinate into spherules.

## DISCUSSION

Here, we define conditions that result in optimal formation of endosporulating spherules. Optimizing *in vitro* spherulation and endospore release will pave the way for in-depth molecular studies of these infection-relevant morphologies. Current antifungals target conserved structures among fungi, but, given their lack of efficacy in treating coccidioidomycosis, pursuing a *Coccidioides*-specific target in the spherule or endospore may prove to be a more effective treatment. In addition, the minimal medium developed here for *in vitro* growth of spherules will enable dissection of the carbon and nitrogen sources that are sufficient for spherule formation and will potentially identify those that are optimal for spherule formation as well. This information could provide insights into host niches that are particularly well suited for spherule growth and persistence and could also lead to the discovery of additional pathways that could serve as antifungal drug targets (43, 44). Given the observation that *Coccidioides* infection can be controlled for long periods of time while a patient is on antifungal treatment but then the infection worsens or relapses upon stopping antifungal treatment (45), it is hypothesized that there is a latent phase of *Coccidioides* infection (46–48). Understanding this latent phase and the alterations in metabolism associated with it could hold the key to definitively curing disseminated infections (49).

Our experiments also explored the role of Tamol in spherulation. Surprisingly, the removal of Tamol from spherulation media revealed a thick visible film surrounding spherules and endospores. *Coccidioides* has been previously observed to shed a lipid-rich outer layer that suppresses fungicidal activity of neutrophils and promotes dissemination of infection (50). We hypothesize that this immunomodulatory shed factor is retained in the absence of Tamol. This observation is reminiscent of the cryptococcal polysaccharide capsule, which is shed and causes immune modulation at a distance (51) or can be retained around cells to play a fungal-protective role (52, 53). Based on these observations, we hypothesize that SOW could play a similar role during *Coccidioide*s infection. Clearly, much more study of SOW is called for, and it may be an excellent antifungal drug target that has yet to be exploited to treat coccidioidomycosis.

Finally, our study of the transcriptome of arthroconidia stocks generated and stored under various conditions indicates the wide variability of cell state for these spore forms. As arthroconidia are the starting material for most experiments in *Coccidioides*, these findings indicate the need to be cognizant of how experiments are designed and that the generation of arthroconidia is an important variable that needs to be controlled to attain reproducible results. While is it likely not feasible to do all experiments with freshly harvested arthroconidia, these data indicate that maintaining consistent duration of storage for comparable samples is important.

## Data Availability

RNA-seq data were deposited in GEO under accession number GSE269000.

## SUPPLEMENTAL MATERIAL

Figure S1 msphere.00679-24-s0001.tiff Spherulation in various media.

Figure S2 msphere.00679-24-s0002.tiff Spherule comparisons across different media types.

Figure S3 msphere.00679-24-s0003.tiff Alterations in morphology and transcriptome caused by storage temperature.

Figure S4 msphere.00679-24-s0004.tiff Viability assay demonstrates variability.

Supplemental material msphere.00679-24-s0005.docx Captions for supplemental figures and tables and final *in vitro* spherulation protocol.

Table S1 msphere.00679-24-s0006.txt Transcript abundance ratios comparing arthroconidia stored at 4°C in PBS for multiple weeks.

Table S2 msphere.00679-24-s0007.txt Transcript abundance ratios comparing arthroconidia generated at 3 different temperatures.

Table S3 msphere.00679-24-s0008.docx Recipe for Converse medium variation.

ASM does not own the copyrights to Supplemental Material that may be linked to, or accessed through, an article. The authors have granted ASM a non-exclusive, world-wide license to publish the Supplemental Material files. Please contact the corresponding author directly for reuse.
