# Supplementary figures and images for: Optimizing in vitro spherulation cues in the fungal pathogen Coccidioides
Source: mSphere. 2024 Dec 17;10(1):e00679-24. doi: 10.1128/msphere.00679-24 (PMC11774042; doi:10.1128/msphere.00679-24)

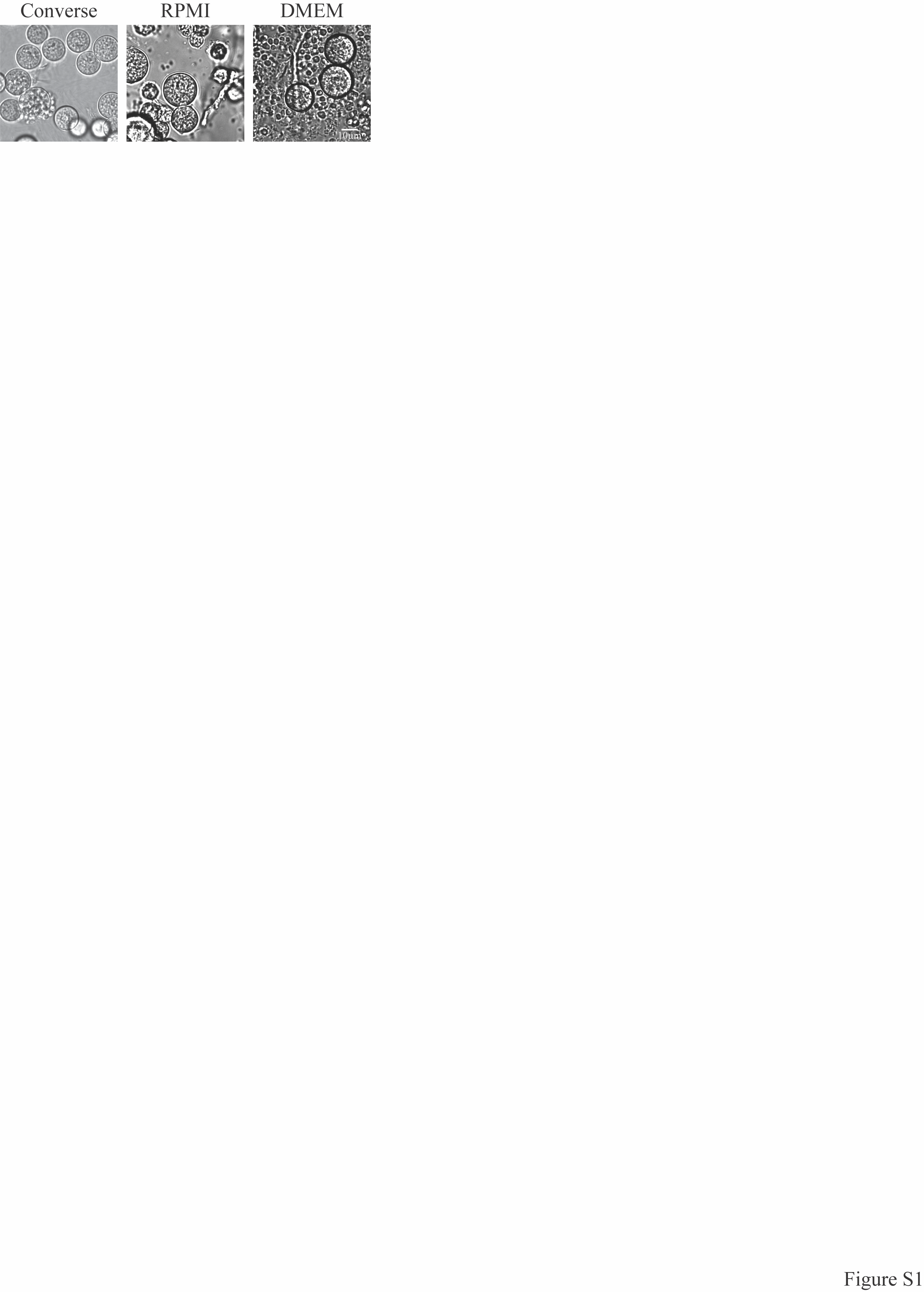

Supplement: Figure S1 — Spherulation in various media. [file msphere.00679-24-s0001.tiff]

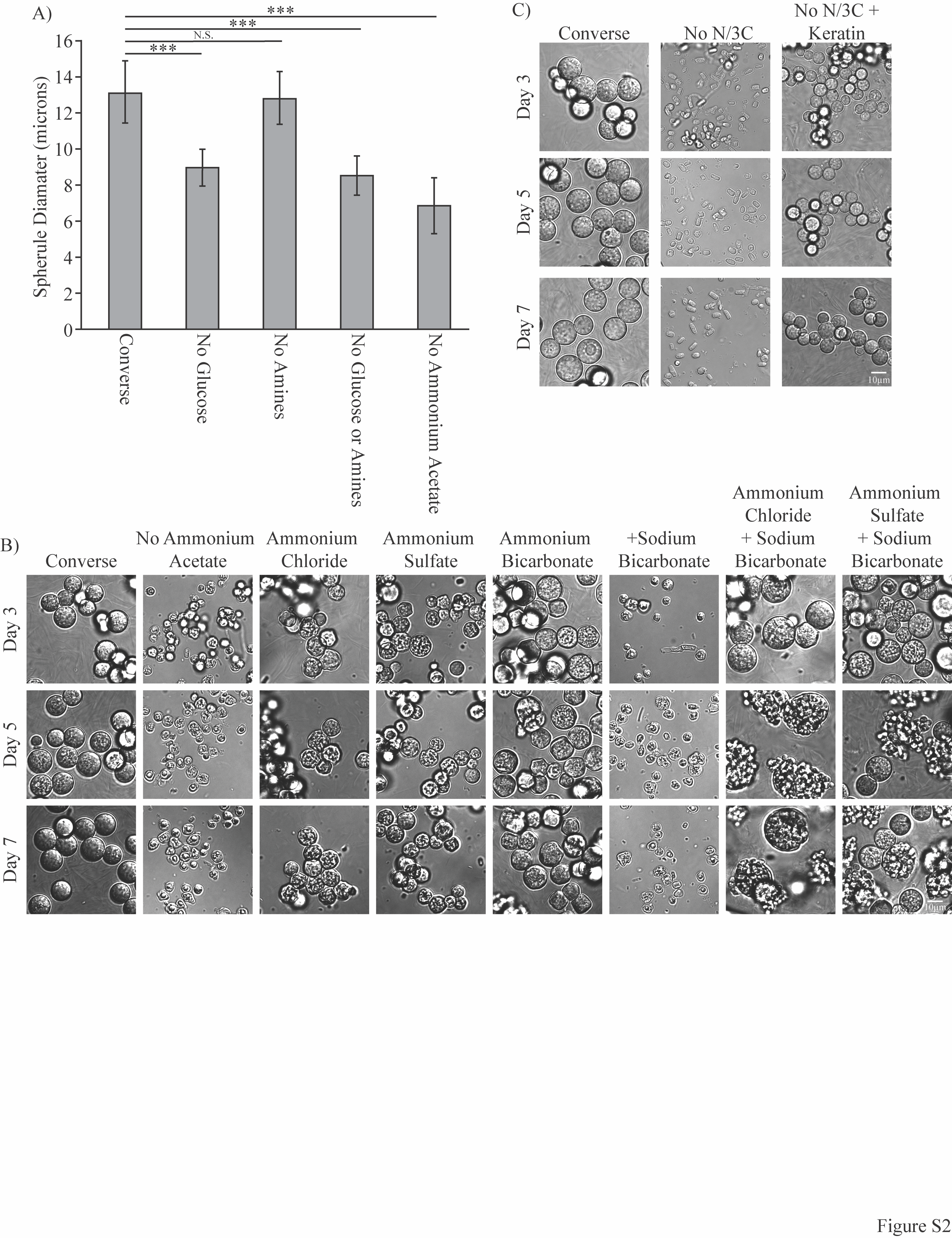

Supplement: Figure S2 — Spherule comparisons across different media types. [file msphere.00679-24-s0002.tiff]

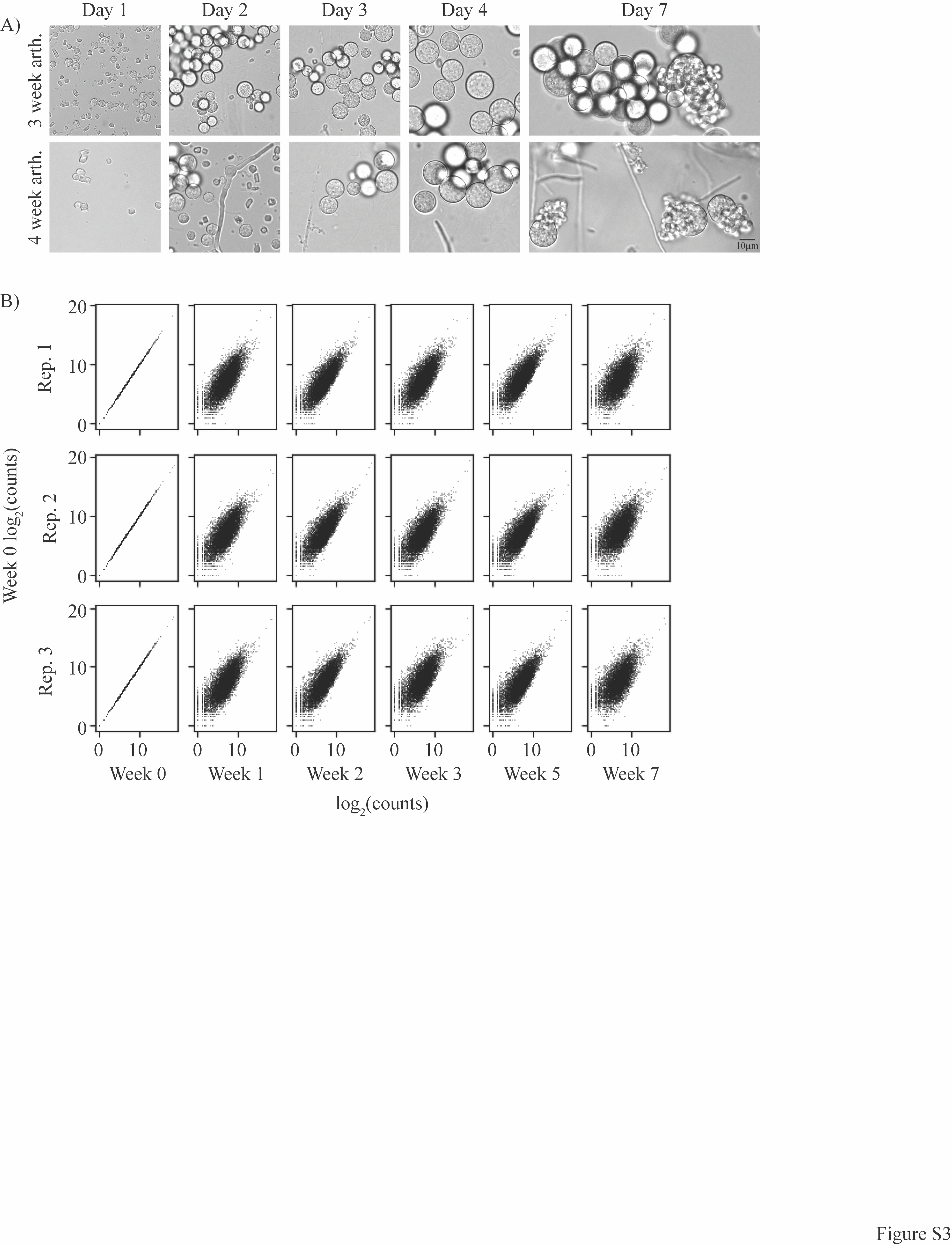

Supplement: Figure S3 — Alterations in morphology and transcriptome caused by storage temperature. [file msphere.00679-24-s0003.tiff]

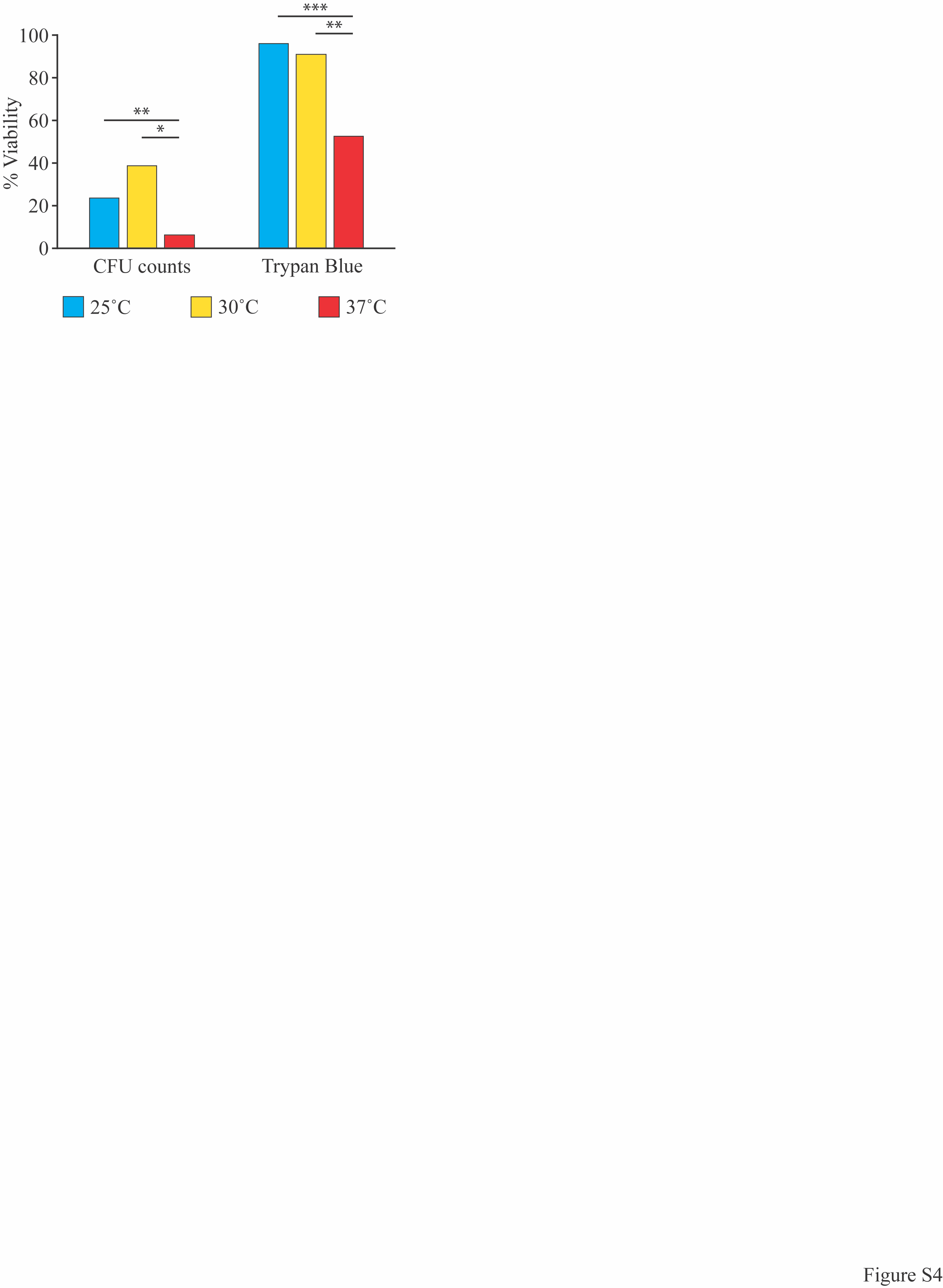

Supplement: Figure S4 — Viability assay demonstrates variability. [file msphere.00679-24-s0004.tiff]
