# Supplementary material for: Optimizing in vitro spherulation cues in the fungal pathogen Coccidioides
Source: mSphere. 2024 Dec 17;10(1):e00679-24. doi: 10.1128/msphere.00679-24 (PMC11774042; doi:10.1128/msphere.00679-24)
Supplement: Supplemental material — Captions for supplemental figures and tables and final in vitro spherulation protocol. [file msphere.00679-24-s0005.docx]

**Supplemental Material**

**Supplemental Figure Captions**

**Figure S1: Spherulation in various media.** 10^6^ arthroconidia/mL were placed in Converse medium + 0.5% Tamol, RPMI + 10% FBS, or DMEM + 20% FBS under standard spherulation conditions (39˚C, 10% CO_2_). Cells were fixed with 4% PFA and images obtained on day 3 of incubation.

**Figure S2: Spherule comparisons across different media types.** A. Average spherule diameter at day 7 for each condition in Figure 2A in microns. Spherule diameters were measured by hand in Fiji for at least 200 spherules per condition. *** p< 0.0001 by 2-sided t-test. 10^6^ arthroconidia/mL were grown in standard spherulation conditions (39˚C, 10% CO_2_) with the media variations described below and monitored for spherule formation by light microscopy on day 3, 5, and 7 post-inoculation. B. Media was either standard Converse, Converse lacking ammonium acetate (No Ammonium Acetate) or Converse in which the ammonium acetate is replaced by the same concentration of ammonium chloride, ammonium sulfate, ammonium bicarbonate, with or without supplemental 0.016 M sodium bicarbonate. C. Media was either Converse or Converse lacking ammonium acetate, glucose, N-Z amines with additional 3% Keratin (No N/3C + Keratin) or without 3% Keratin (No N/3C).

**Figure S3: Alterations in morphology and transcriptome caused by storage temperature**. A. Arthroconidia stored at 4˚C in PBS for 3 and 4 weeks were placed in standard spherulation conditions and fixed cells were monitored by light microscopy at day 1, 2, 3, 4, and 7 post-inoculation. Arthroconidia that had been stored for longer amounts of time exhibited more hyphal contamination of spherule cultures. B. Scatterplots of log_2_(counts) for each gene in freshly harvested Week 0 arthroconidia (y axis) versus arthroconidia stored for 1-7 weeks in PBS at 4˚C (x axis), demonstrating large-scale transcriptomic changes are not due to a normalization artifact.

**Figure S4: Viability assay demonstrates variability.** Percent of viable arthroconidia assessed by trypan blue exclusion (on day of arthroconidia harvest) or by CFU counts (after growth at 30˚C for 72h) in independently-generated spore stock replicates from 6A. These replicate data demonstrate variability in this assay. * p<0.05, ** p<0.005, *** p<0.0005 by paired t-test.

### Table S1: Tab-delimited text file containing transcript abundance ratios comparing arthroconidia stored at 4˚C in PBS for multiple weeks

Each row corresponds to a transcript. The columns are as follows: UNIQID: systemic gene name from Silveira genome (12). Systematic gene names with _1, _2 appended have multiple isoforms as detected by kallisto although not all isoforms passed read count filter. NAME: Short gene name from Mandel et al (14). Cp_anno: GenBank Cp Silveira annotation. CiRS: systematic CiRS gene name for the InParanoid-mapped *C. immitis* RS ortholog. CiRS_anno: Genbank annotation for CiRS ortholog. HcG217B: systematic HcG217B GSC gene name for the InParanoid-mapped *Histoplasma* G217B ortholog. HcG217B_anno: GSC annotation for HcG217B ortholog. The next 5 columns give limma adjusted p-values for differential expression for the listed contrasts. The next 18 columns give kallisto estimated normalized counts for each sample. The next 5 columns give limma generated log_2_ fold change values for the listed contrasts.

### Table S2: Tab-delimited text file containing transcript abundance ratios comparing arthroconidia generated at 3 different temperatures

Each row corresponds to a transcript. The columns are as follows: UNIQID: systemic gene name from Silveira genome (12). Systematic gene names with _1, _2 appended have multiple isoforms as detected by kallisto although not all isoforms passed read count filter. NAME: Short gene name from Mandel et al (14). Cp_anno: GenBank Cp Silveira annotation. CiRS: systematic CiRS gene name for the InParanoid-mapped *C. immitis* RS ortholog. CiRS_anno: Genbank annotation for CiRS ortholog. HcG217B: systematic HcG217B GSC gene name for the InParanoid-mapped *Histoplasma* G217B ortholog. HcG217B_anno: GSC annotation for HcG217B ortholog. The next 3 columns give limma adjusted p-values for differential expression for the listed contrasts. The next 18 columns give kallisto estimated normalized counts for each sample (6 replicates per temperature condition). The next 3 columns give limma generated log_2_ fold change values for the listed contrasts.

**Table S3: Recipe for Converse medium variation**

1. Make 100mL each of the above stocks in ddH_2_O (sterile filter and store at 4˚C)
2. Put the indicated volumes of each stock (righthand column) into 600mL of ddH_2_O
3. Add 5g of Tamol powder
4. Add 4g of dextrose powder
5. Adjust pH to 6.5 with 1N NaOH or 1N HCl
6. Bring to 1 L total volume with ddH_2_O
7. Sterile filter.

**Optimized in vitro spherule growth protocol:**

Materials:

Converse medium variation adapted from 11 (See Table S3)

# 125mL screwtop polypropylene flasks (Nalgene 4109-0125)

Quantified freshly-harvested *Coccidioides* arthroconidia stock (per protocol published in 11).

1. Place 50 mL Converse medium into plastic 125mL polypropylene screwtop flasks.
2. Calculate the volume of arthroconidia stock required to inoculate 50 mL of medium at 10^6^ arthroconidia/mL.
3. Enter BSL3 and prepare biosafety cabinet to work with *Coccidioides.*
4. Vortex tightly-closed arthroconidia stock for 30 seconds in biosafety cabinet.
5. Transfer appropriate amount of arthroconidia stock to each flask. Carefully and snugly close each flask after inoculating them.
6. Disinfect each closed flask and remove from biosafety cabinet.
7. Dry flasks thoroughly with paper towels.
8. Place flasks in incubator. Once flasks are secured in incubator, loosen screw cap by one quarter turn to allow air exchange, and grow at 39˚C, 10% CO_2_, 120 rpm.
