# Supplementary material for: Optimizing in vitro spherulation cues in the fungal pathogen Coccidioides
Source: mSphere. 2024 Dec 17;10(1):e00679-24. doi: 10.1128/msphere.00679-24 (PMC11774042; doi:10.1128/msphere.00679-24)
Supplement: Table S3 — Recipe for Converse medium variation. [file msphere.00679-24-s0008.docx]

| Reagent | Stock concentration | Volume / 1L Converse |
| --- | --- | --- |
| Ammonium Acetate | 1.596 M | 10 mL |
| KH_2_PO_4_ anhydrous | 0.37 M | 10 mL |
| K_2_HPO_4_ anhydrous | 0.3 M | 10 mL |
| MgSO_4_7H_2_O | 0.16 M | 10 mL |
| ZnSO_4_7H_2_O | 2.5 mM | 5 mL |
| NaCl | 24 mM | 10 mL |
| CaCl_2_2H_2_O | 4.08 mM | 5 mL |
| NaHCO_3_ | 14.3mM | 10 mL |
| N-Z amines | 5g / L | 10 mL |
